# Normal orbit skeletal changes in adolescents as determined through cone-beam computed tomography

**DOI:** 10.1186/s13005-016-0130-0

**Published:** 2016-11-10

**Authors:** B. Lee, C. Flores-Mir, M. O. Lagravère

**Affiliations:** 1 Department of Dentistry, University of Alberta, Edmonton, Canada; 2 Department of Medicine and Dentistry, School of Dentistry, University of Alberta, 5524 Edmonton Clinic Health Academy, 11405-87 Ave, Edmonton, T6G 1C9 Canada

**Keywords:** Orbit, Cone-beam computed tomography, Growth, Orthodontics

## Abstract

**Background:**

To determine three-dimensional spatial orbit skeletal changes in adolescents over a 19 to 24 months observation period assessed through cone-beam computed tomography (CBCT).

**Methods:**

The sample consisted of 50 adolescents aged 11 to 17. All were orthodontic patients who had two CBCTs taken with an interval of 19 to 24 months between images. The CBCTs were analyzed using the third-party software Avizo. Sixteen anatomical landmarks resulting in 24 distances were used to measure spatial structural changes of both orbits. Reliability and measurement error of all landmarks were calculated using ten CBCTs. Descriptive and *t*-test statistical analyses were used to determine the overall changes in the orbits.

**Results:**

All landmarks showed excellent reliability with the largest measurement error being the Y-coordinate of the left most medial point of the temporalis grooves at 0.95 mm. The mean differences of orbital changes between time 1 and time 2 in the transverse, antero-posterior and vertical directions were 0.97, 0.36 and 0.33 mm respectively. Right to left most antero-inferior superior orbital rim distance had the greatest overall transverse change of 4.37 mm. Right most posterior point of lacrimal crest to right most postero-lateral point of the superior orbital fissure had the greatest overall antero-posterior change of 0.52 mm. Lastly, left most antero-inferior superior orbital rim to left most antero-superior inferior orbital rim had the greatest overall vertical change of 0.63 mm.

**Conclusions:**

The orbit skeletal changes in a period of 19–24 months in a sample of 11–17 year olds were statistically significant, but are not considered to be clinically significant. The overall average changes of orbit measurements were less than 1 mm.

## Background

The orbit is a complex structure composed of seven bones. These bones include the frontal, lacrimal, ethmoidal, maxillary, zygomatic, sphenoid and palatine bones [1]. The orbit itself is considered to be a four-walled unit where each wall has its own clinically important structures [2]. Due to the orbit’s association with the eye globe and surrounding structures, proper understanding of orbital growth can be beneficial to different fields of medicine and dentistry. Applications include preoperative planning for orbit reconstruction, orbital rehabilitation to promote normal orbital growth, forensic identification and orthodontic diagnosis and treatment planning [3–5]. The latter because of the concept that intraorbital measurements are presumed to be stable after 8 years of life and can be used as reference structures when assessing craniofacial changes.

Escaravage and Dutton have previously done computed tomography (CT) analyses of the orbit and have determined that orbital growth is highly influenced by globe growth [4]. Their findings suggested that orbital growth occurs more significantly during the first 2 years of life and especially during the first year of life [4]. Furthermore, their findings suggested a steady pace that carries on until growth parameters reach 85–90 % of their adult size around 8 years of life [4]. Since the pace of orbital growth slows past 8 years of life, further growth analyses would be beneficial to determine the stability of the orbits in later years for orthodontic diagnosis and treatment planning as mentioned before.

Imaging is a necessary diagnostic tool in the practice of orthodontics. Traditionally, most intra-oral and extra-oral radiographic imaging was done by means of two-dimensional (2D) radiography until the recent introduction of cone-beam computed tomography (CBCT). The use of CBCT in orthodontic practices have been made possible due to the low radiation doses compared to medical CT, short image acquisition times, low cost and relatively high image quality [6]. Furthermore, three-dimensional (3D) radiography has advantages over 2D radiography as it is not limited by distortion, magnification, superimposition and misrepresentation of structures experienced with 2D projections [7]. With this type of imaging, clinicians and researchers are able to analyze the orbit structural change and stability over time in 3D. This would help clinicians identify if the orbit still presents changes during the adolescent and adult years. Therefore, the purpose of this study was to determine 3D spatial orbit skeletal changes in adolescents over a 19 to 24 months observation period assessed through CBCT.

## Methods

This study was approved by an institutional review board. Images used were obtained retrospectively from a previous clinical trial where CBCT scans were used to measure three-dimensional changes produced by normal growth and orthodontic treatment changes. The sample was taken from 50 patients aged 11 to 17. All individuals were developmentally normal and no gross anatomical abnormalities were identified. Each patient had CBCTs taken within a time interval of 19–24 months. The inclusion criteria for the participants were to have full permanent dentition, non-syndromic characteristics nor previous craniofacial surgery. Patients should all be present in the age range of 11–17 years of age. Any patient not following the previous criteria was excluded.

CBCT scans were taken using the ICat New Generation (Imaging Sciences International, Hatfield, USA) machine at 0.3 mm voxel size and 8.9 s (Large field of view 16cmx13.3 cm, 120kVp, 18.54mAs) and converted into DICOM format. AVIZO software (Visualization Sciences Group, Massachusetts, USA) was used to analyze the DICOM format images. Sagittal (YZ-plane), coronal (XZ-plane), and axial (XY-plane) volumetric slices, as well as 3D reconstructions of the images were used to determine 16 landmarks located in the cranial base and skeletal orbits. The analysis was done by the main researcher (BL) having been trained previously by ML who has extensive experience and publications in terms of CBCT landmarking.

**Fig. 1:**
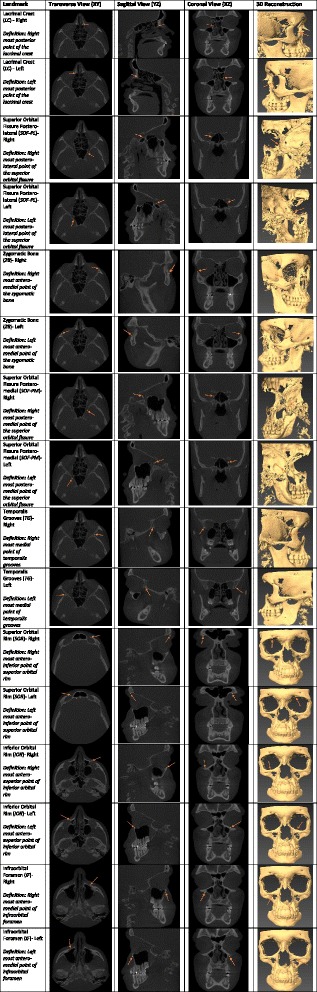
Landmark definition and visual representation on cross-sectional and 3D images

Each landmark was recorded as a point with X, Y and Z values in the Cartesian coordinate system. The investigator was blind to the patient’s age and time when the CBCT was taken. Landmarks were located using both 3D reconstructions and 2D slices. Definitions of the landmarks located are listed in Fig. 1.

By using these landmarks, 24 distances were used to assess changes of the orbits through comparison of two time-deferred data sets of each patient. These distances (Table 1) assessed growth of the orbits through measuring transverse, antero-posterior and vertical spatial and structural changes of the orbits. Linear distances (d) between landmarks were analyzed using the XYZ coordinates in the following equation.

**Table 1:** Overall change of the distances between T1 and T2 in the transverse, antero-posterior and vertical dimensions

Distances (mm)	Greatest overall change	
	Mean	Std. deviation
Transverse		
Right *LC* to Left *LC*	0.37	1.76
Right *SOF-PL* to Left *SOF-PL*	0.80	0.92
Right *ZB* to Left *ZB*	1.13	2.54
Right *SOF-PM* to Left *SOF-PM*	0.53	0.96
Right *TG* to Left *TG*	0.91	2.47
Right *SOR* to Left *SOR*	4.37	2.48
Right *IOR* to Left *IOR*	4.35	2.42
Right *IF* to Left *IF*	0.83	0.96
Right *LC* to Right *ZB*	0.39	2.14
Left *LC* to Left *ZB*	0.41	2.30
Right *SOF-PL* to Right *SOF-PM*	0.14	0.81
Left *SOF-PL* to Left *SOF-PM*	0.13	1.12
Antero-posterior		
Right *LC* to Right *SOF-PL*	0.52	1.32
Left *LC* to Left *SOF-PL*	0.45	1.38
Right *ZB* to Right *SOF-PM*	0.46	1.08
Left *ZB* to Left *SOF-PM*	0.50	1.27
Right *ZB* to Right *TG*	0.50	2.01
Left *ZB* to Left *TG*	0.13	1.98
Right *TG* to Right *SOF-PM*	0.03	1.67
Left *TG* to Left *SOF-PM*	0.27	1.69
Vertical		
Right *SOR* to Right *IOR*	0.42	1.79
Left *SOR* to Left *IOR*	0.63	1.78
Right *IOR* to Right *IF*	0.17	2.55
Left *IOR* to Left *IF*	0.38	2.02

$$ d=\sqrt{{\left({X}_1-{X}_2\right)}^2+{\left({Y}_1-{Y}_2\right)}^2+{\left({Z}_1-{Z}_2\right)}^2} $$

To test examiner reliability in terms of consistent landmarking, a reliability trial was performed separate from the study trials. The 16 landmarks were identified from each image on three occasions at different times, for ten randomly selected images from ten different individuals. Intra-examiner reliability values were determined using intraclass correlation coefficients (ICCs). Landmark definitions and procedures were the same as those used in the study.

Twenty-four linear distances were obtained for each CBCT image. Distances were selected to cover all possible orientations and dimensions without being repetitive. Descriptive statistics were calculated for all distances and for the differences between corresponding distances at the two time points. Sex and age distribution are shown in Table 2. All distances were then analyzed using paired *t*-test to verify statistical significance (*P* < 0.05). Distances were also grouped dependent on their orientation and a univariate analysis of variance followed by Bonferroni post-hoc test was done to verify any statistical significance between dimensional changes.

**Table 2:** Sex and age distribution

Category	Average age (Years)
Males (18)	13.6 + - 2.7
Females (32)	14.7 + - 2.5
Total (50)	14.2 + - 2.5

## Results

Overall measurement errors of all landmarks in the study were determined by analyzing ten CBCT data sets that were chosen randomly. The largest measurement error was in the Y-coordinate of the left most medial point of the temporalis grooves at 0.95 mm. The smallest measurement error was the x-coordinates of both the right most antero-inferior point of the superior orbital rim and left most antero-inferior point of the superior orbital rim at <0.01 mm. The lowest ICC value was from the right most antero-inferior point of the superior orbital rim and the left most antero-inferior point of the superior orbital rim with ICC values of 0.99 (C/I 0.96–1.00). Given the smallest and largest measurement errors, all landmarks have excellent reliability. Table 3 shows all the landmarks and their respective measurement errors.

**Table 3:** Mean X, Y, Z coordinate measurement errors for each landmark

Landmark		Mean	Std. deviation
Lacrimal Crest (LC)- Right	X	.3091	.43438
	Y	.4303	.43550
	Z	.0909	.10445
Lacrimal Crest (LC)- Left	X	.1758	.27370
	Y	.3576	.44649
	Z	.0909	.10445
Superior Orbital Fissure Postero-lateral (SOF-PL)- Right	X	.3576	.43642
	Y	.3091	.33236
	Z	.0909	.10445
Superior Orbital Fissure Postero-lateral (SOF-PL)- Left	X	.5770	.25629
	Y	.1515	.39787
	Z	.1030	.12424
Zygomatic Bone (ZB)- Right	X	.3394	.32722
	Y	.2121	.29936
	Z	.0909	.10445
Zygomatic Bone (ZB)- Left	X	.4121	.38852
	Y	.1152	.22329
	Z	.0909	.10445
Superior Orbital Fissure Postero-medial (SOF-PM)- Right	X	.3879	.45978
	Y	.3758	.33635
	Z	.0909	.10445
Superior Orbital Fissure Postero-medial (SOF-PM)- Left	X	.1412	.26373
	Y	.3212	.44703
	Z	.0909	.10445
Temporali s Grooves (TG)- Right	X	.2061	.30178
	Y	.7939	.77198
	Z	.0909	.10445
Temporalis Grooves (TG)- Left	X	.2667	.32249
	Y	.9515	.75548
	Z	.0909	.10445
Superior Orbital Rim (SOR)- Right	X	0.0000	0.00000
	Y	.0333	.04714
	Z	.4667	.56569
Superior Orbital Rim (SOR)- Left	X	0.0000	0.00000
	Y	.3000	.42426
	Z	.3000	.14142
Inferior Orbital Rim (IOR)- Right	X	.1000	.14142
	Y	.1000	.14142
	Z	.2000	.18856
Inferior Orbital Rim (IOR)- Left	X	.1000	.14142
	Y	.4667	.28284
	Z	.4667	.18856
Infraorbital Foramen (IF)- Right	X	.2606	.32449
	Y	.0909	.20715
	Z	.0545	.09342
Infraorbital Foramen (IF)- Left	X	.3733	.32933
	Y	.0667	.16600
	Z	.0545	.09342

By using the 24 distances obtained from the 16 landmarks, we calculated the differences between time 1 and time 2 and correlated the changes to transverse, antero-posterior and vertical directions. After applying a multivariate analysis, age and sex did not have a statistical significant effect on the measurement changes obtained. Therefore those variables were not further considered in the follow up statistical analysis. When applying the univariate analysis of variance, the orientation of the distances did present a statistical significant difference in terms of change (*P* < 0.05). When applying the Bonferroni post-hoc test, the transverse dimension showed to be statistically significantly different to the other dimensions (*P* < 0.05). Table 4 shows the mean differences of orbital changes between T1 and T2 in the transverse, antero-posterior and vertical directions as 0.97 mm (*P* < 0.05), 0.36 mm (*P* < 0.05) and 0.33 mm (*P* > 0.05) respectively. As a result, the transverse and antero-posterior changes are statistically significant. Given that the changes were less than 1 mm for the transverse, antero-posterior and vertical directions, the changes should not necessarily be considered clinically significant. The greatest overall transverse change was 4.37 mm which occurred with the right to left most antero-inferior superior orbital rim distance. The greatest overall antero-posterior change was 0.52 mm which was observed with the right most posterior lacrimal crest to right most postero-lateral superior orbital fissure. Lastly, the greatest overall vertical change was 0.63 mm and was from the left most antero-inferior superior orbital rim to left most antero-superior inferior orbital rim.

**Table 4:** Mean difference of orbital change between T1 and T2 in the transverse, antero-posterior and vertical dimensions

Direction (mm)	Paired differences		
	Mean	Std. deviation	Sig. (2-tailed)
Transverse	0.97205	2.24565	0.000
Antero-posterior	0.35879	1.54073	0.000
Vertical	0.32636	2.04374	0.071

## Discussion

CBCT imaging has been gaining wide acceptance in recent years due to its low radiation exposure, low imaging costs and high spatial resolution [6]. Given its recent acceptance in the field of dentistry, research in the area of CBCT has not been as vast as compared to CT. This is largely due to the fact that CT scanners were invented in 1972 by Hounsfield and Cormack, while the first commercial CBCT dental unit was not introduced to Europe until 1999 [8]. One disadvantage present of CBCT vs. CT is the distinction between soft tissues having CT presenting a higher contrast [8, 9]. In terms of orbit growth, selecting the landmarks to be used to measure changes in the orbit can be difficult. Escaravage and Dutton’s study [4] based on CT analyses of the orbit largely influenced our study. Landmarks such as the most posterior point of the lacrimal crest, most antero-medial points of the zygomatic bone and the most medial points of the temporalis grooves were also analyzed in our study. With our 16 landmarks and corresponding 24 distances, we were able to comprehensively analyze 3D spatial orbit skeletal changes in adolescents through CBCT imaging.

It is worth noting that landmark identification is a major source of measurement error. These errors arise in the process of identifying specific landmarks with factors including sharpness of the radiographic images, landmark definition, human error and procedural errors [10, 11]. Three-dimensional imaging has been shown to greatly reduce projection errors compared to traditional two-dimensional imaging. Thus, in the process of selecting adequate landmarks, all these possible errors should be taken into account [12].

**Fig. 2:**
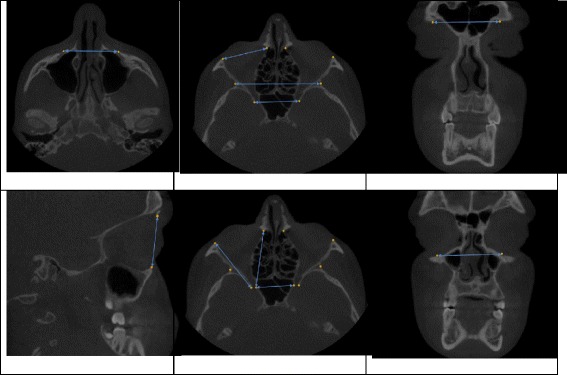
Diagrams illustrating some of the distances used to assess Transverse, Antero-posterior and Vertical Dimensions

To better analyze the results of our study, we decided that grouping the landmark distances in terms of their dimensional orientation, on a three-space plane orientation, could allow us to better visualize growth patterns. Given the fact that we were unable to find other publications as references which tried this measurement approach, we hypothesized that assessed distances in the transverse, antero-posterior and vertical measurements present the most logical sequence. Our interpretations included twelve, eight and four distance measurements for the transverse, antero-posterior and vertical dimensions respectively (Fig. 2). The mean differences of orbital changes between T1 and T2 in the transverse, antero-posterior and vertical dimensions were 0.972, 0.359 and 0.326 mm respectively.

The greatest overall change of all the distances was found to be the right to left most antero-inferior superior orbital rim and antero-superior inferior orbital rim at 4.37 and 4.35 mm respectively. Given that changes in all other distances were less than 1 mm, it could be hypothesized that these landmarks were influenced by normal growth. The right to left most antero-inferior and antero-superior orbital rim were closely located to where the frontal sinus ends. Since the ICC values showed that these landmarks are reliable, it is possible that the frontal sinus is not entirely stable during adolescent growth. It has been previously reported that the frontal sinus undergoes significant growth changes well into adolescence. This is supported by Ruf and Pancherz’s study [13] which showed that frontal sinus growth velocity has a large variation intra- and inter-individually. Pubertal peaks were determined to exist at a mean of 1.9 mm/year and cessation of frontal sinus growth could not be determined as sinus growth has been seen to finish at the end of pubertal growth while in other cases growth exceeded the skeletal maturity stage. Another study [14] reported that frontal sinus development is completed by age 18 with increased expansion mostly in length until age 8 and between years 12 to 14 were not statistically associated with the study findings.

### Limitations

The small study sample size can be considered a limitation and our results can be interpreted as preliminary findings.

Additional limitations revolve around the difficulty of determining landmarks due to the quality of some of the CBCT data sets in terms of noise and slice orientation. Landmarks of particular difficulty in terms of identification and pinpointing were the posterior lacrimal crest, infraorbital foramens, superior and inferior orbital rim. Due to the quality of certain CBCT data sets, omissions occurred for some landmarks due to the inability to accurately define them in the given data sets. Furthermore, since slice angulations were based off of the individual’s positioning while taking the CBCT, some CBCT data sets were not used in cases where the slice orientations were heavily skewed in order to limit inaccuracies.

A change in the CBCT machine (from NewTom 3G - Aperio Services, Verona, Italy at 110 kV, 6.19mAs and 8 mm aluminum filtration, voxel size of 0.3 mm) used in the graduate orthodontic program also occurred between some T1 and T2 data sets and should be considered as a potential limitation to consistency. This occurred in only three of the cases so the real impact is likely unimportant.

Another limitation was the fact that although we grouped the distances in terms of dimensional orientation, the distances are not entirely in 2D of transverse, antero-posterior or vertical as they contain 3D coordinates. These limitations need to be addressed in the future direction of our study along with the inclusion of additional angular measurements and volumetric changes of the orbit based off of the existing landmarks.

## Conclusion

The orbit skeletal and spatial changes were statistically significant, but should not normally be considered clinically significant. The overall average orbit dimensional changes were less than 1 mm. Our results show that the orbits should be considered a good structure to be used for superimposition since in this sample there were minimal, but not clinically relevant, changes during the 19 to 24 months observation period among patients aged between 11 and 19 years.

## Abbreviations

2D: Two-dimensional
3D: Three-dimensional
CBCT: Cone-beam computed tomography
CT: Computed tomography
ICC: Intraclass correlation coefficients
